# Cenderitide-Eluting Film for Potential Cardiac Patch Applications

**DOI:** 10.1371/journal.pone.0068346

**Published:** 2013-07-04

**Authors:** Xu Wen Ng, Yingying Huang, Horng H. Chen, John C. Burnett, Freddy Y. C. Boey, Subbu S. Venkatraman

**Affiliations:** 1 School of Materials Science & Engineering, Nanyang Technological University, Singapore, Singapore; 2 Division of Cardiovascular Diseases and Internal Medicine, Mayo Clinic, Rochester, Minnesota, United States of America; University of California, San Diego, United States of America

## Abstract

Cenderitide, also known as CD-NP, is a designer peptide developed by combining native mammalian c-type natriuretic peptide (CNP) and the C-terminus isolated from the dendroapis natriuretic peptide (DNP) of the venom from the green mamba. In early studies, intravenous and subcutaneous infusion of cenderitide was reported to reduce left ventricular (LV) mass and ameliorate cardiac remodelling. In this work, biodegradable polymeric films encapsulating CD-NP were developed and were investigated for their in vitro release and degradation characteristics. Subsequently, the bioactivity of released peptide and its effects on human cardiac fibroblast (HCF) were explored. We achieved sustained release from three films with low, intermediate and high release profiles for 30 days. Moreover, the bioactivity of released peptide was verified from the elevated production of cyclic guanosine monophospate (cGMP). The CD-NP released from films was able to inhibit the proliferation of hypertrophic HCF as well as suppress DNA synthesis in HCF. Furthermore, the sustained delivery from films showed comparable or superior suppressive actions on hypertrophic HCF compared to daily infusion of CD-NP. The results suggest that these films could be used to inhibit fibrosis and reduce cardiac remodelling via local delivery as cardiac patches.

## Introduction

Post myocardial infarction (MI), if intervention is not carried out immediately, excessive necrosis occurs in the myocardium. To preserve the cardiac output, the heart undergoes massive functionality and morphological changes and left ventricular (LV) remodelling is triggered. LV remodelling is a compensatory mechanism where the ventricular chamber dilation and wall thinning occurs [1]–[6]. These changes result in the loss of contractile function, decreased output and ultimately congestive heart failure (HF) [3], [5]–[7].

Currently, ventriculoplasty surgeries are the most widely performed procedures to counter LV remodelling in hospitals. Ventriculoplasty such as the Dor procedure involves the reduction of chamber size and management of dilation alone [8]; these do not adequately halt fibrosis progression and are invasive [3]. Although cardiovascular therapeutics like angiotensin-converting enzyme (ACE) inhibitors, beta-blockers and aldosterone antagonist [9], [10] administered concomitantly showed beneficial effects in slowing down the progression of CHF, however, the mortality and morbidity of patients with CHF remained high. Moreover, common side effects including dizziness and low blood pressure have made them unsatisfactory. In cardiovascular treatment of LV remodelling, the lack of the less invasive procedures and appropriate therapeutic agent are major problems.

The human recombinant natriuretic peptides (NPs) [11]–[15] had been singled out as a new-age cardiovascular therapeutic agent, particularly for their role in acute decompensated HF [16]–[18] and ventricular remodelling [19]–[22] treatment. However, there are limitations, such as atrial natriuretic peptide (ANP) and brain natriuretic peptide (BNP) are hypotensive in nature and c-type natriuretic peptide (CNP) lacks the desired renal effects [21], [23], [24]. To achieve the benefits while minimizing the detrimental effects of different peptide, Mayo Clinic has developed CD-NP [19], [25]. CD-NP is a chimeric peptide produced from the fusion of c-type NP (CNP) and the C-terminus of dendroaspis NP (DNP) [13], [26] isolated from the venom of a green mamba. Burnett and co-workers have shown that intravenous and subcutaneous infusion of CD-NP reduced LV mass in MI-model rodents, exhibited cardiac unloading in dogs [19] and induced natriuretic and blood pressure responses in humans [20]. These studies suggest that CD-NP possesses potent anti-fibrotic properties desired in attenuating cardiovascular pathologies associated with collagen accumulation post MI.

However, the clinical use of CD-NPs had been largely hindered by its short elimination half-life (18.4±1.4 minutes), delicate nature and the absence of suitable administration routes. Currently, the means of delivering NPs have been via intravenous or subcutaneous infusion [14], [16], [17], [27]–[30]. However, IV infusions have to be done in a hospital setting, continuously for several days or weeks following MI; the subcutaneous administration is performed via the implantation of a pump, which requires hospital visits for implantation/removal and it is also uncomfortable to the patient. Moreover, such systematic delivery is low in efficacy as pathology is not targeted locally.

In this paper, we postulate that the development of a cenderitide-eluting platform could enable the local and targeted delivery of CD-NP to the site of need to provide more efficient treatment. This paper is divided into 3 main parts. In the first part, we focus on film development, in vitro CD-NP release and film morphology and degradation characterization. In the second part of this study, we attempt to understand CD-NP’s inhibitory effects on in vitro human cardiac fibroblast (HCF) cells. Finally, we evaluate the effects of these CD-NP releasing films on HCF particularly focusing on the retention of bioactivity of encapsulated CD-NP and sustain effects from released platforms.

## Materials and Methods

### 1. Materials

CD-NP and Human Cardiotrophin-1 (CT-1) were obtained from the American Peptide Company and GenWay (USA) respectively. Poly (ε-caprolactone) (PCL) (Mn: 80,000 g/mol) was purchased from Sigma-Aldrich (USA). Phosphate buffer solution (PBS), pH 7.4 was purchased from OHME scientific. All organic solvents were of analytical grade and were used as received.

### 2. Sample Preparation

The polymer solution was prepared by dissolving 0.9 g PCL pellets in 5 mL of dichloromethane (DCM) and stirred continuously to obtain homogenous solution. The peptide solution was prepared separately, by dissolving 1 wt% of CD-NP in water (150 µL) or ethanol (1000 µL) according to their co-solvent system formulation. Subsequently, the polymer solution and peptide solution were added together and emulsified, where the emulsification duration was monitored. Finally, the solution was solvent cast on glass panels via an automatic film applicator. The films were left in room temperature ambience overnight before relocating to the 37°C vacuum oven for 7 days to remove any residual solvent. The residual solvent was verified to be less than 1 wt% using the thermogravimetric analyzer (TGA, TA Instruments Q500).

To obtain different release rates, 3 biodegradable polymeric films encapsulating CD-NP were developed using different co-solvent systems at varied emulsification conditions. Film 1 was prepared using the water/DCM co-solvent system, emulsified for 10 minutes. Film 2 was prepared using the ethanol/DCM, where the effect of emulsification duration was negligible. Film 3 was prepared using the water/DCM co-solvent system, emulsified for 1 hour. Table 1 gives a summary of the manufacturing condition and initial release classification of the formulations.

**Table 1:** Summary of formulation information.

Film	Initial release	Co-solvent system	Emulsification duration	CD-NP loading
1	Slow	water/DCM	10 minutes	1 wt%
2	Intermediate	ethanol/DCM	10 minutes/1 hour	1 wt%
3	Fast	water/DCM	1 hour	1 wt%

Formulation manufacturing condition and initial release classification.

### 3. In vitro Release Study

Samples were cut into 1 cm × 1 cm and prepared in triplicate. The release study was carried out by immersing samples in PBS in 37°C incubator to mimic the physiological conditions. At pre-determined time-points, the PBS was entirely replenished to maintain sink condition. And PBS containing the released peptide was tested using the micro-bicinchoninic acid protein assay (Pierce). The samples were prepared in accordance to the manufacturer’s protocol and the absorbance detection was tested at 526 nm wavelength using the UV-Vis spectrophotometer (UV- 2501, Shimadzu).

### 4. In vitro Degradation and Mass Loss

The degradation study was carried out by immersing the films in PBS and replenished at pre-determined time-points. At the pre-determined time-points, samples were rinsed with deionized water and dried in 37°C vacuum oven for 1 week before characterization.

The degree of degradation was monitored via the changes in molecular mass which was measured using the gel permeability chromatography (GPC, Agilent series 1100). And mass loss was determined by comparing the initial and final masses of films at each time-point and the calculation for percentage mass loss is shown in the following equation.
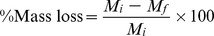



### 5. Surface Morphological

Samples were coated with gold for 20 seconds using the gold sputter machine under argon atmosphere. Initial and post immersion surface morphology were viewed using the scanning electron microscopy (SEM) (AS Jeol 6360) at 3 kV.

### 6. In vitro Human Cardiac Fibroblast (HCF) Culture

Human cardiac fibroblast (HCF) cells were purchased from lonza. Cells were grown and maintained using fibroblast growth medium (FGM, Lonza) consisting of recombinant human insulin, r-Human fibroblast growth factor-B (rhFGF-B), gentamicin sulfate amphotericin-B (GA-100) and 10% fetal bovine serum (FBS). Cells were cultured in atmosphere 5% CO_2_ in a 37°C incubator. Only cells from passage 3 to 5 were used for this experiment.

### 7. Detection of 3′, 5′ Cyclic Guanosine Monophospate (cGMP)

The intracellular cGMP production was detected using the cGMP assay (GE Health). 8,000 cells were seeded in 48-well plate for 24 hours. The cells were incubated with peptides in PBS (without Magnesium and Calcium) containing 10 mM of HEPES, 0.1% FBS and 0.5 mM 1-methyl-3-isobutyl xanthine at condition of pH 7.4 at 37°C for 30 minutes. The reaction was terminated by aspirating the reagents and the cells underwent lysis using the *lysis regent* given in the cGMP kit. Finally, cells were tested in accordance with the manufacturer’s protocol using the Tekan microplate reader.

### 8. Cell Viability

Cell viability was monitored using the Real-Time Cell Analyzer dual-plate (RTCA DP) Instrument, xCELLigence system (Roche Applied Science). The system measures the real–time cellular activities via electrical impedance detected from tissue culture E-plates that consist of integrated gold micro-electrodes at the bottom of the plates. The system measures the changes in impedance and converts them into a dimensionless parameter known as Cell Index (CI). The CI is calculated by the following equation 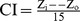, where Zi and Zo represent the individual point and background measurement at the commencement of the experiment respectively. The change in impedance occurs due to change in number of cells attachment or due to dimensional changes of attached cells.

First, 50 µL of culture medium was added to determine the background of the E-plates. Next, 100 µL of HCF cell suspension was added (8,000 cells/well) and allowed to settle before addition of another 50 µL of culture medium. In the pre-conditioning step, cells were grown, conditioned in serum free medium and stimulated with CT-1 for 20–24 hours each sequentially. The impedance was monitored hourly during the entire preconditioning. For daily dose treatment groups, cell culture plate was removed and replenished with CD-NP daily. For film groups, film samples were prepared as 0.25 cm × 1 cm long strips that lined against the culture plate wall and fully immersed but not touching the cells. The impedance of both the daily dose and CD-NP film treatment groups were monitored hourly, the CI values were calculated and plotted against time on graph. Additionally, relative cell index (RCI), a mean of comparing treatment group with respect to control group was calculated to be 
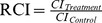
, where RCI <1 denotes inhibition of cell viability.

### 9. DNA Synthesis

The bromo-2′-deoxyuridine BrdU assay (Roche) was used to detects DNA synthesis. 8,000 cells were seeded in 96-well plate and allowed to grow for 24 hours prior treatment. Cells underwent serum-deprivation for 24 hours to induce quiescence. Quiescent cells were incubated with CT-1 for 24 hours. Following this stimulation, CD-NP was added into HCF every 24 hours. Next, cells were labelled with the BrdU label for 24 hours and measured at absorbance 370 nm. Relative DNA synthesis of tested groups was normalized against the control (no addition of CD-NP) groups, where the control groups were set as the reference.

### 10. Statistical Analysis

Results are presented in mean ± standard deviation. The one-way analysis of variance (ANOVA) was used to compare significant difference and p<0.05 is denoted as statistically significant.

## Results

### 1. In vitro Release from Films

**Figure 1:**
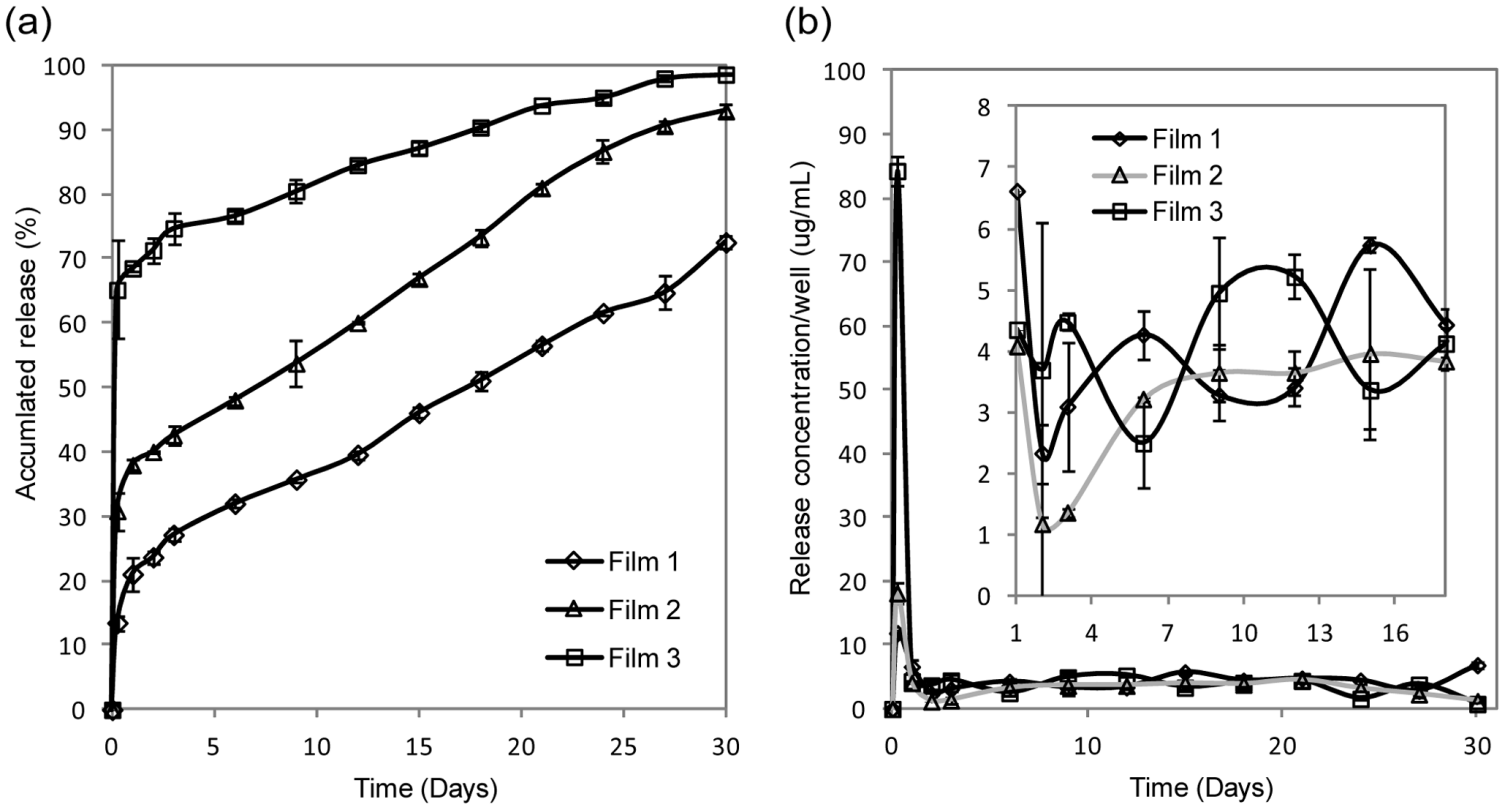
Accumulated release profiles of CD-NP loaded films for 30 days. (a) Accumulated peptide release profiles of film 1, 2 and 3 and (b) bottom left graph shows the release concentrations of film 1, 2 and 3 over 30 days; top right graph is a zoomed in on the bottom left graph.

From figure 1a, film 1 and film 3 had the lowest and highest initial (burst) release of 13% and 65% respectively. Subsequently, film 1 and film 3 released 60% and 99% CD-NP by 30 days. Film 2 had an intermediate initial burst release of 31% and released 93% of CD-NP at the end of 30 days. In figure 1b, the concentration of the CD-NP (following the burst release) from all 3 films were more or less similar from 1 to 30 days (in the range of 1–6 µg/mL).

### 2. In vitro Degradation and Mass Loss

**Figure 2:**
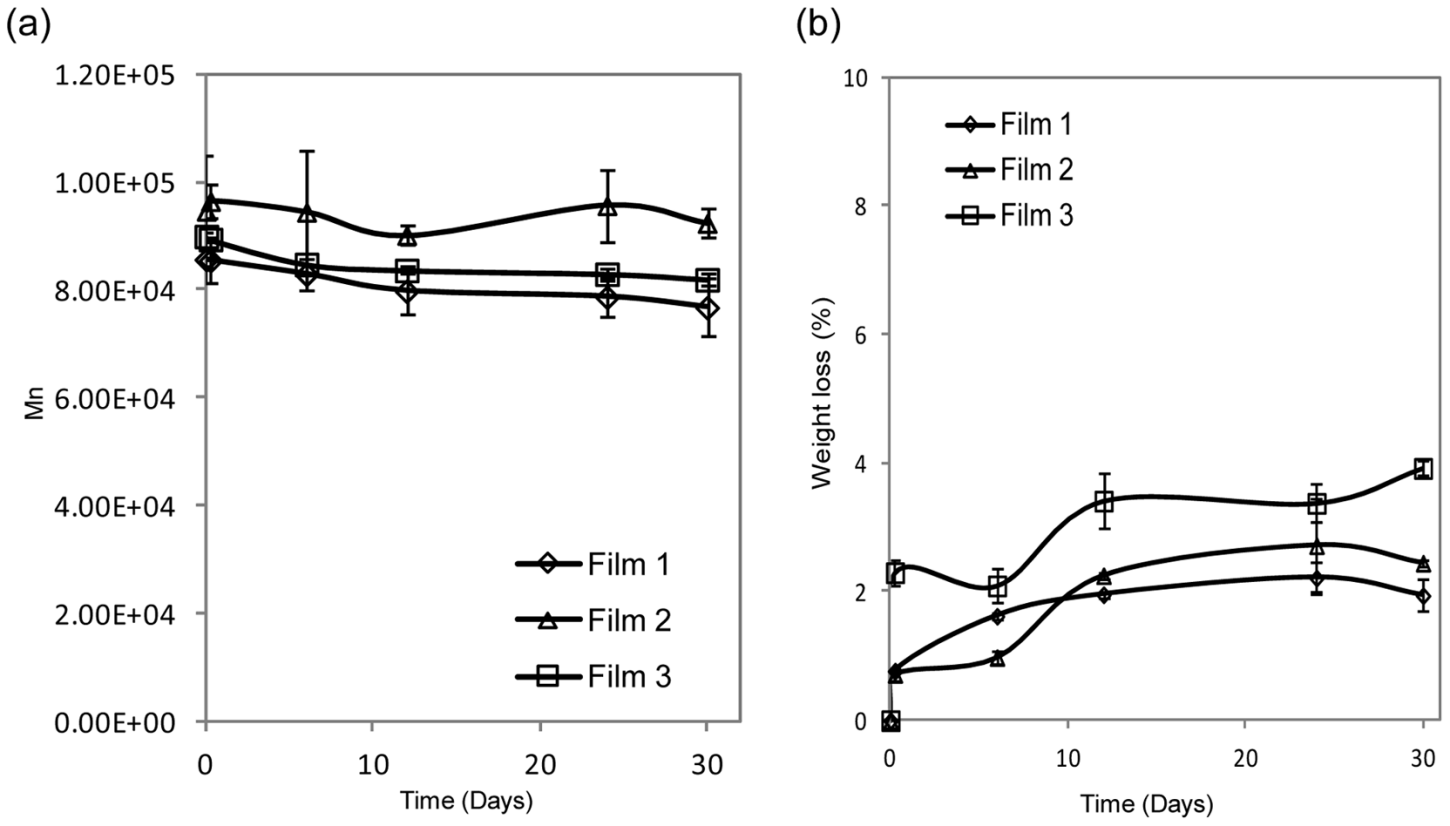
Molecular mass and mass loss of CD-NP loaded films. (a) Molecular mass change and (b) mass loss of CD-NP loaded film 1, 2 and 3 over 30 days.

The degradation of the films was determined by measuring the molecular mass (figure 2a) and total mass (figure 2b) changes. There was no significant molecular mass change and mass loss in all three tested films, indicating the slow degradation of PCL.

### 3. Surface Morphology

**Figure 3:**
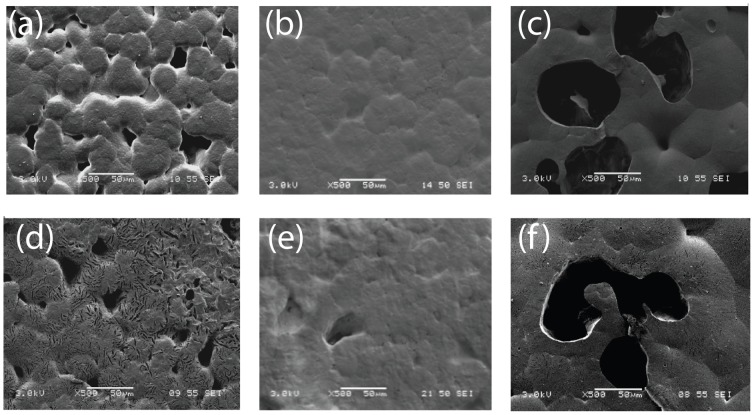
Surface morphology of films loaded with CD-NP. SEM micrograph on day 0 of CD-NP loaded (a) film 1, (b) film 2 and (c) film 3 and after day 30 release in (d) film 1, (e) film 2 and (f) film 3.

Figure 3a, b and c present the initial surface morphology of films 1, 2 and 3 respectively. Both film 1 and film 3 appear to be more porous compared to film 2, which may be due to the use of an immiscible co-solvent system. And by using a longer period of emulsification, film 3 appears to be more porous compared to film 1. Figure 3d, e and f show the surface morphology of films 1, 2 and 3 after 30 days of immersion. The overall integrity of the 3 films after 30 days was maintained. Micro- cavities were found in films 1 and 3, which was absent in film 2.

### 4. Effects of CD-NP on 3′,5′ Cyclic Guanosine Monophosphate (cGMP) Production

**Figure 4:**
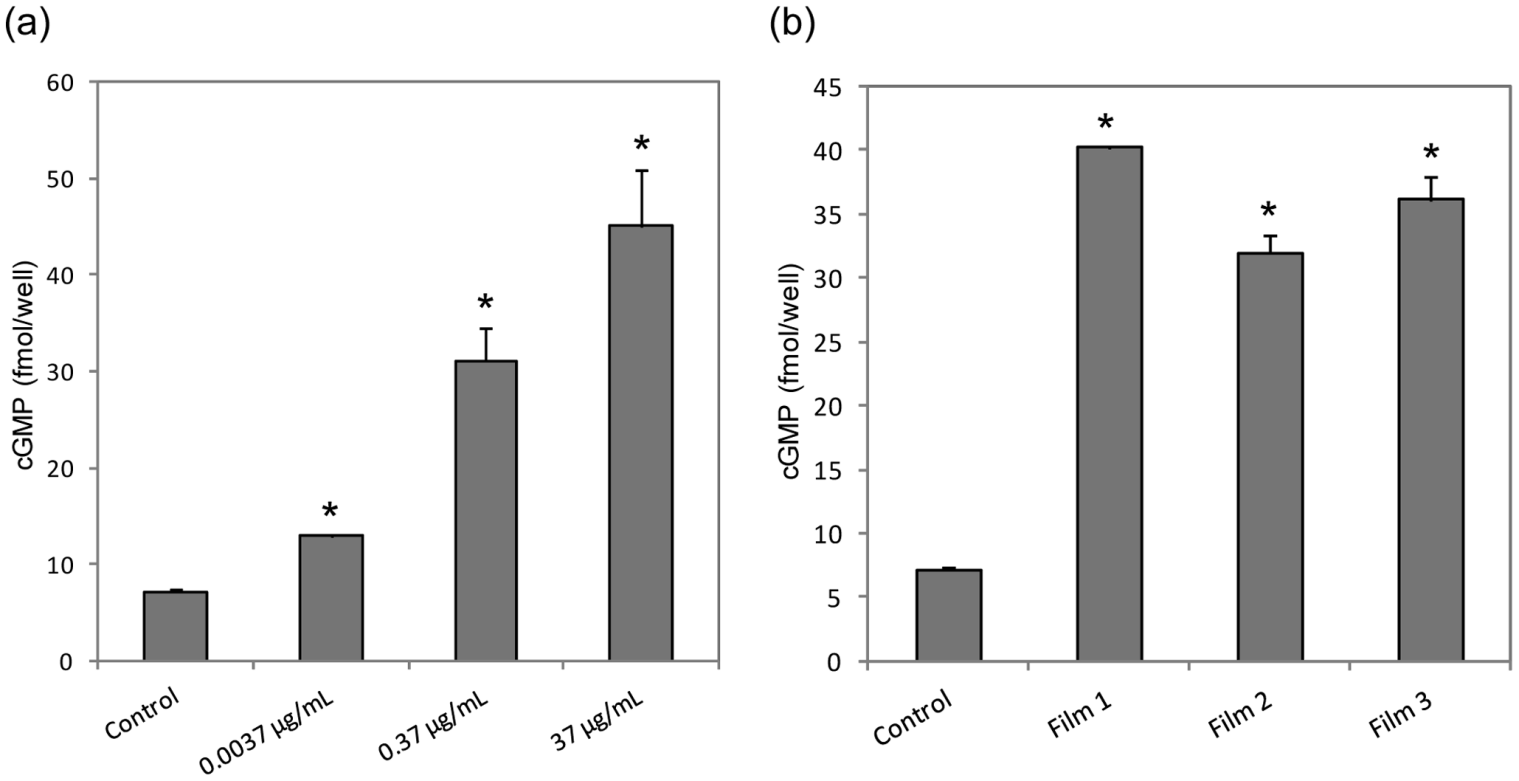
Cyclic 3′5′ guanosine monophosphate (cGMP) generation in human cardiac fibroblast (HCF). cGMP generation in HCF induced by (a) different CD-NP concentration and (b) 24 hour peptide released from film 1, 2 and 3, *p<0.05 versus control.

3′,5′ cyclic guanosine monophosphate (cGMP) is a secondary messenger produced when natriuretic peptides bind to the GC receptors. In figure 4a, the addition of CD-NP to HCF cells led to elevation in cGMP level. Different CD-NP concentrations were added and a dose dependent relationship within the concentration range of 0.0037 µg/mL to 37 µg/mL was observed. The ANOVA test of significance was carried out to compare between 37 µg/mL and 0.37 µg/mL, where the 100-fold difference in concentration resulted in statistically-significant differences in cGMP levels (p<0.05) However, it should be noted that there is an absence of statistical significance between 10-fold difference in concentrations, such as between 3.7 µg/mL and 37 µg/mL (results not shown).

To verify if CD-NP encapsulated in the films retained bio-activity, CD-NP released at the 24 hour time-point was investigated. In figure 4b, the cGMP production levels after the addition of released CD-NP from all three films were elevated significantly compared to the control group (p<0.05).

### 5. Effects of CD-NP on Human Cardiac Fibroblast (HCF) Cell Viability

**Figure 5:**
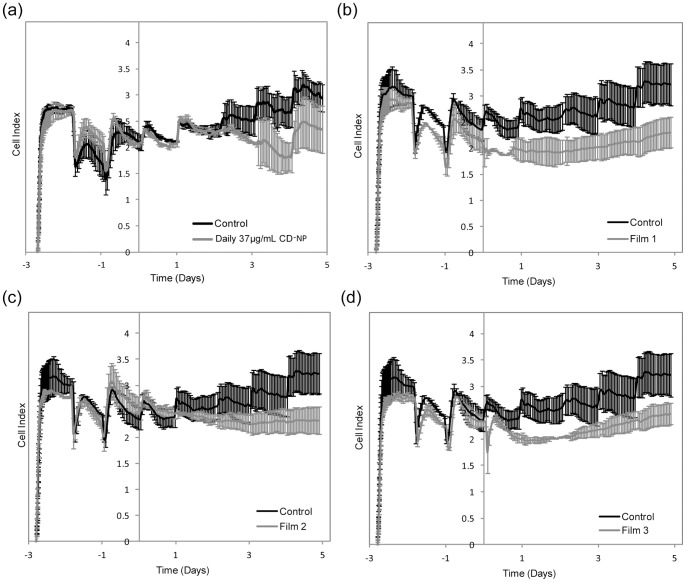
Cell Index (CI) measurements. Cell Index (CI) measurement of control compared to (a) Daily infusion of CD-NP, (b) film 1, (c) film 2 and (d) film 3 from the RTCA xCELLigence.

In figure 5, the graphs of cell index (CI) of HCF against time is presented, where CI increment denotes increase in cell proliferation or cell spreading. Figure 5a shows the cell viability of HCF after daily dose of 37 µg/mL CD-NP compared to control. It can be seen that in the first 48 hours, there was no distinct difference between the CD-NP group and control, however, a downward trend started to develop after the 3^rd^ dose was administered. By the addition of the 4^th^ dose (figure 5a), it was clear that daily dose of CD-NP at concentration of 37 µg/mL resulted in lower CI compared to control. Figure 5b, c, d shows the cell viability study of HCF of films 1, 2 and 3 respectively against the control group. Both film 1 and 3 showed immediate decline of CI compared to control, whilst, film 2 only saw decline in CI compared to control on the 4^th^ day.

**Figure 6:**
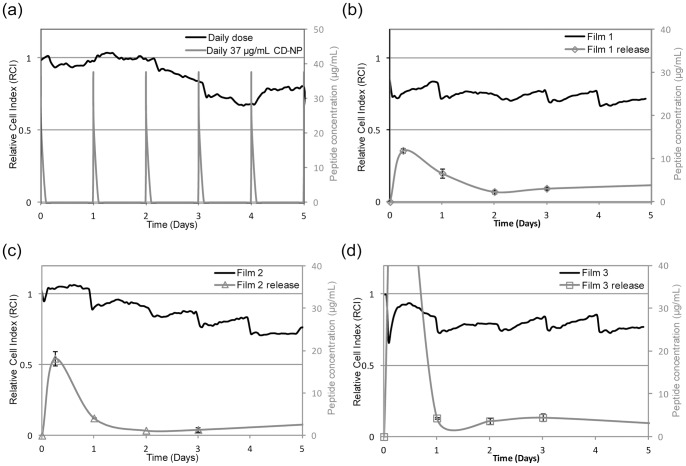
Correlation between relative cell index (RCI) and CD-NP concentration. Correlation between RCI (primary y-axis) and peptide concentration (secondary y-axis) of (a) Daily infusion of CD-NP, (b) film 1, (c) film 2 and (d) film 3 over 5 days (x-axis).

The relative cell index (RCI) is used to describe the cell viability in a comparative manner, where lower the RCI value denotes greater extent of inhibition. Figure 6a b, c and d shows the correlation between the RCI (primary y-axis) and peptide concentration (secondary y-axis) with respect to time. From figure 6a, we can see that daily dosing of CD-NP results in “spikes” of CD-NP to 37 µg/mL daily (secondary y-axis), but the RCI was only less than 1 on the 2^nd^ day onwards. Films 1 and 3 had RCI value less than 1 from 0 to 5 days. Film 2 however only saw RCI less than 1 after the 1^st^ day. By the 5^th^ day, all three films had RCI less than 1.

### 6. Effects of Released CD-NP of HCF Cells

**Figure 7:**
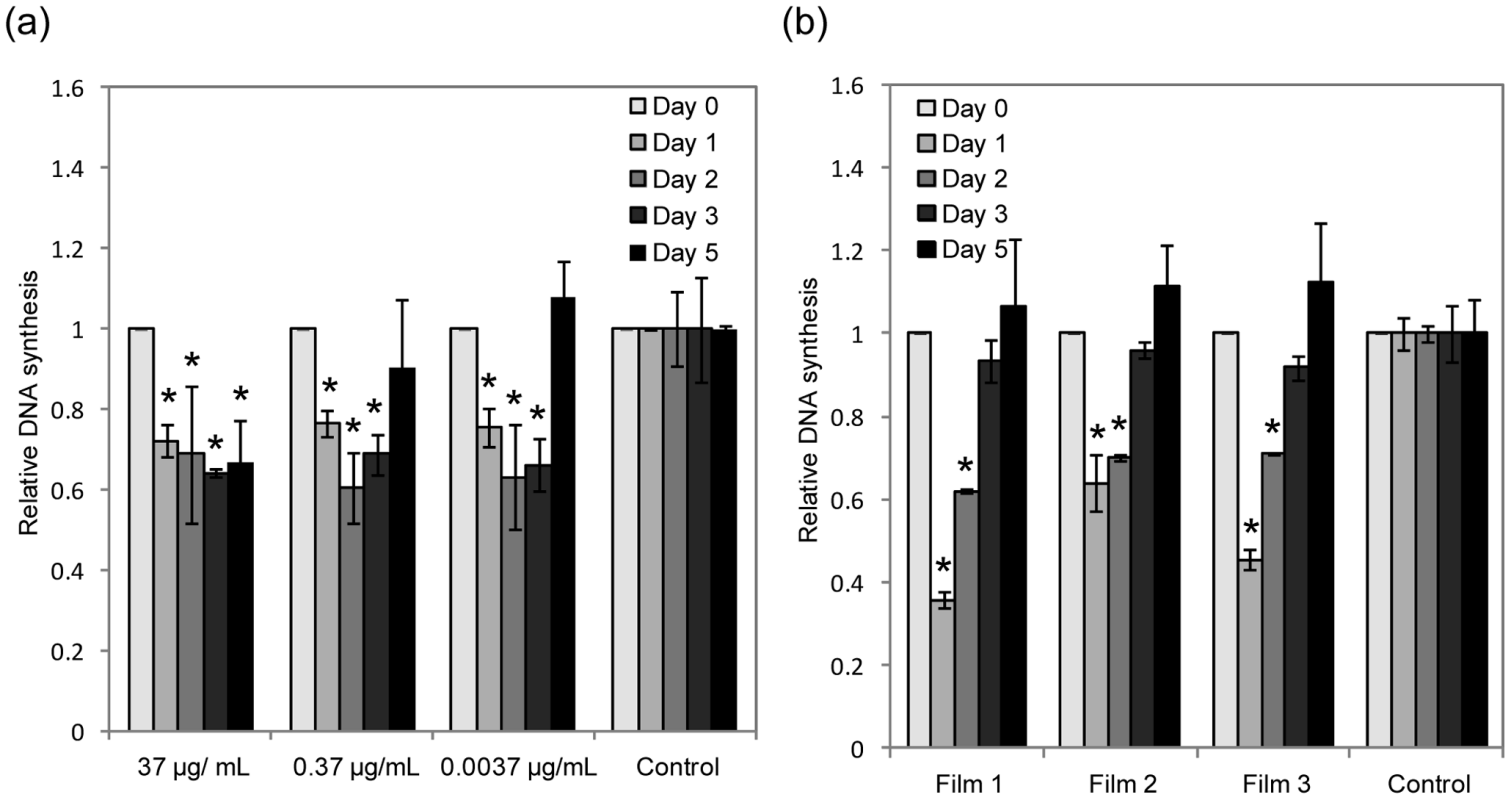
Effects of CD-NP on human cardiac fibroblast (HCF). Relative anti-proliferation actions of (a) CD-NP of different concentration and (b) CD-NP released from film 1, 2 and 3 (1 day, 2 days, 3 days and 5 days) in HCF via colormetric bromodeoxyuridine (BrdU), *p<0.05.

To understand the effects of CD-NP addition on cellular proliferation at the DNA level, the DNA synthesis in HCF were investigated. Figure 7a shows the relative DNA synthesis of the addition of CD-NP of different concentrations compared to control. CD-NP between the ranges of 0.0037 µg/mL to 37 µg/mL showed suppression in the synthesis of DNA. No concentration dependence was observed between CD-NP concentration and the amount of DNA synthesized. Furthermore, the daily dose of 37 µg/mL of CD-NP showed that DNA synthesis could be suppressed up to 5 days. Lower concentrations of CD-NP (0.37 µg/mL and 0.0037 µg/mL) suppressed DNA synthesis only up to 3 days.

Figure 7b shows the effect of different CD-NP releasing films on the relative DNA synthesis in HCF. All films showed statistically significant suppression of DNA synthesis up to 2 days. Although all three films appear to show lower DNA synthesis on the 3^rd^ day, it was not statistically significant.

## Discussion

LV remodelling results in the loss of contractile functions, deterioration of cardiac function and eventually death as a resultant of HF [6]. For patients with end-stage HF, heart transplantation is the best option but only a minority of the patients benefit due to the limited number of donor hearts available. Although ventricular surgical procedures are widely practised, their clinical outcome remains unsatisfactory due to the limited regenerative ability of the matured heart [2]. The placement of an anti-fibrotic-eluting cardiac patch to prevent fibrotic scar development is a promising strategy to reverse LV remodelling.

The current work presents the development of polymeric matrix that provides sustained release of CD-NP. Sustained release of CD-NP from three different initial release profiles of high, medium and low up to 30 days was attained. Additionally, the bioactivity of released cenderitide was verified through the inhibition of HCF proliferation studies and the elevation of intracellular cGMP in HCF. Finally, the performance of CD-NP released from polymer films were compared to daily dose CD-NP in their inhibition of hypertrophic and hyperplasia HCF. Our results seemed to indicate that continuous delivery may be necessary for optimal inhibition of hypertrophic HCF.

CD-NP is a relatively new therapeutic entity; the therapeutic window has not been fully established. From the literature, the CD-NP infusion range between 0.1–30 ng/kg/minute (for 1–7 days) and 10–30 ng/kg/day (for 5–30 days) via intravenous and subcutaneous infusions were reported to be effective; however there is no literature on an ideal therapeutic profile [20]. Hence when developing the films, we explored different release profiles while keeping in mind the effective concentrations reported. This led us to select 3 distinctly different initial release profiles. All three films achieved sustained CD-NP release for up to 30 days with two phase release profiles. We believe that the first phase release is attributed to the immediate dissolution of CD-NP found near or at the surface of the film. As we observed no significant film degradation from our degradation study, we believe that subsequent release over 30 days was due to diffusion controlled release of hydrophilic CD-NP diffusing out of hydrophobic polymer matrix. The slow degrading characteristics of PCL [31], [32]and the diffusion release from PCL [33] had been reported in literature respectively. All films displayed sustained release over time in the range of 12–84 µg/mL (for 0–6 hours) and 1–6 µg/mL (for 1–30 days). Daily subcutaneous infusion of CD-NP at 10–30 ng/kg/day over period of 5 to 30 days had successful elevation of plasma and urine cGMP whilst improving cardiac load and minimizing arterial pressure [20]. Our film (1 cm × 1 cm × 0.004 cm) was able to achieve release of 1–6 ng/kg/day (between 1 to 30 days), suggesting that it could potentially be developed into cardiac patches, which are likely to be larger in volume for treatment purpose.

Polymeric PCL was chosen as material for film development because it is biocompatibility, elastic mechanical properties and predictable biodegradability [31], [32]. As a cardiac patch or ventricular restrain device, the slow degrading nature of PCL is advantageous because the temporal presence of it post MI could act as mechanical support preventing recurrent LV remodelling, whilst its eventual degradation precludes potential complications associated with non-degradable materials [2], [34].

cGMP is a secondary messenger molecule generated when the GC receptor is stimulated by NP. In the literature, CD-NP had been reported to exert anti-fibrotic actions and regulate homeostasis through the elevation of cGMP. In our study, CD-NP elicited elevation of cGMP production in a dose dependent manner as expected. Upon establishing the relationship of CD-NP and cGMP production, we tested the CD-NP release from the films to verify the retention of bioactivity. The CD-NP released from all three films showed elevation of cGMP, implying the retention of bioactivity. After verifying that CD-NP elicits cGMP production, we moved on to understand the inhibition effects of CD-NP on CT-1 induced HCF using two methods.

Fibrosis is a process involving disproportionate accumulation of fibrillar collagen, stiffening of ventricles and eventual impairment of ventricular contraction and relaxation [1], [2], [4], [7], [35]. Since cardiac fibroblast is responsible for producing extra-cellular matrix (ECM) proteins, it is apparent that inhibiting the fibroblast cells would be a “nip-in-the-bud” approach to prevent collagen accumulation [35], [36]. During fibrosis, secretion of cytokines induces the accumulation of fibrillar collagen and impairs the ventricular contraction and relaxation of the LV. In particular, CT-1 directly stimulates pathological hypertrophy and induces chamber dilation in both in vivo animal studies and in vitro cardiac fibroblasts [6], [36], [37]. From the xCELLigence data, HCF treated with CT-1 observed an increase in CI, which implies that HCF was successfully stimulated to spread and proliferate. The daily dose of CD-NP on CT-1 induced HCF was investigated and inhibition of HCF commenced after the 3^rd^ dose. Moreover, pronounced inhibition was observed after the 5^th^ dose, implying that multiple dosing is essential for effective inhibition.

Next, the films were investigated; films 1 and 3 exhibited early and sustained inhibitory effects, this suggests that the performance of a sustained supply of lower CD-NP concentration surpassed that of a daily higher concentration supply. This observation could be attributed to the short elimination half-life of CD-NP (18.4±1.4 minutes), where each administered dose only had brief biological effects [25]. Both films 1 and 3 displayed almost immediate and sustained inhibition over 5 days, indicating that the inhibitory effect was independent of the high or low initial release. Film 2 had an intermediate initial release yet, there was an absence of inhibition in the beginning. The results seem to hint that CD-NP encapsulated in water/DCM system might have more superior bio-activity compared to the ethanol/DCM system. Such arguments had also been previously reported, where water is less harsh compared to organic solvents, provides hydration and does not implicate any toxicity issues. These properties make water an ideal co-solvent for the encapsulation of proteins and peptides [38]. One may argue that no statistically significant difference was detected between the cGMP elevated by CD-NP released from water/DCM and ethanol/DCM systems. However, the fact that only 100-fold difference in concentration could be picked up by the cGMP assay indicates that a more sensitive technique may be needed to resolve this discrepancy.

The second method used to investigate the inhibitory effect of CD-NP on HCF was by means of elucidating amount of DNA synthesis in HCF as a consequence of the presence of CD-NP. DNA synthesis of HCF was suppressed in presence of CD-NP. The suppression was independent of dose (37 µg/mL, 0.37 µg/mL and 0.0037 µg/mL) for the first 3 days of daily dose. However, by the 5^th^ day, 0.37 µg/mL and 0.0037 µg/mL were rendered ineffective in inhibiting the DNA synthesis of HCF. This observation suggests that long-term dose responsiveness differs from short-term dose responsiveness, which maybe associated to the interdependence of GC receptor pathways and NP exposure [30], [39]. The suppression of DNA synthesis was observed from CD-NP releasing films only for the first 2 days. The absence of suppression on the 5^th^ day was expected as the critical dose was not met. However, the lack of DNA suppression on the 3^rd^ day was unexpected. Since the CD-NP released from the films was within the working range (0.0037 µg/mL to 37 µg/mL). Two hypotheses to explain this observation are proposed here. Firstly, the lack of DNA synthesis suppression could be attributed to the different types of exogenous CD-NP profiles. The daily dosing of CD-NP has an “On-off” (bolus) profile (figure 6), whilst film release had an ‘on’ profile throughout the entire duration. These two distinct profiles may elicit different cascading modes and biological responses; resulting in different DNA synthesis behaviour. The second explanation is that the encapsulated CD-NP had become less potent. Although this is undesirable, it may be inevitable due to the manufacturing processes [38], [40].

The xCELLigence method is unable to differentiate whether hypertrophic or hyperplasia HCF was inhibited. On the other hand, the inhibition of HCF cell hyperplasia was determined by the DNA synthesis study. When we correlate both data, we observed that when CD-NP suppressed DNA synthesis, similar inhibition of HCF was not observed in the xCELLigence experiment and vice versa. This suggests that the inhibition observed in xCELLigence is dominantly the inhibition of hypertrophic HCF cells. From the CD-NP released from films 1 and 3, continuous inhibition of HCF was observed from xCELLigence despite the disappearance of DNA synthesis suppression by the third day. This means that continuous supply of CD-NP eluting from films were more effective in inhibiting hypertrophic HCF compared to “on-off” profile of daily dose of CD-NP.

Post infarct LV remodelling could be broadly divided into the early phase and late phase. Early phase remodelling occurs within the first 72 hours and dominantly involves the expansion of the infarct zone [6]. During this phase, if the secretion of modified extracellular matrix collagen by activated fibroblast is not halt, the transition of granulation tissue to scar tissue becomes permanent. From our study, films 1 and 3 exhibited early inhibition on hypertrophic HCF compared to film 2. Hence, films 1 and 3 could be considered in further studies for tackling early phase remodelling. However, further long-term investigation needs to be carried out if late phase remodelling is considered; this is because more complicated factors including the decrease in collagenase activity and upregulation in the secretion of collagen entails time-dependent LV dilation. Particularly, further in vivo studies are needed to elucidate CD-NP’s effect on early and late phase remodelling.

The peptide-eluting cardiac patch was proposed with the intention to be implanted via minimally invasive deployment methods [41]–[44]. Current ventriculoplasty surgeries serve to reinforce the LV mechanically but do not provide any functional benefits in preventing progression of disease or in restoring contractile functions of a failing heart. The implantation of a CD-NP-releasing cardiac patch or ventricular device using minimally invasive procedures could provide a mechanical barrier to counteract accelerated LV dilation and minimize fibrotic scar formation via the elution of anti-fibrotic CD-NP and counter cardiac remodelling.

### Conclusion

The successful encapsulation and release of bioactive CD-NP from films was achieved. And sustained release of 3 release profiles of high, medium and low initial release were attained up to 30 days. This work demonstrated that the cenderitide eluted from polymeric platform was more effective in suppressing hypertrophic HCF compared to daily dose. The eluted CD-NP inhibited the proliferation of both hypertrophic and hyperplasia HCF, indicating that it could be potentially used for treatments of cardiac remodelling pathologies.
